# Overcoming the Slow Recovery of MOX Gas Sensors through a System Modeling Approach

**DOI:** 10.3390/s121013664

**Published:** 2012-10-11

**Authors:** Javier G. Monroy, Javier González-Jiménez, Jose Luis Blanco

**Affiliations:** 1 Department of System Engineering and Automation, University of Málaga, Campus de Teatinos, 29071 Málaga, Spain; E-Mail: javiergonzalez@uma.es; 2 Department of Engineering, University of Almería, 04120 La Cañada, Almería, Spain; E-Mail: joseluisblancoc@gmail.com

**Keywords:** metal oxide semiconductor sensor, mobile robotic olfaction, gas sensing, electronic nose

## Abstract

Metal Oxide Semiconductor (MOX) gas transducers are one of the preferable technologies to build electronic noses because of their high sensitivity and low price. In this paper we present an approach to overcome to a certain extent one of their major disadvantages: their slow recovery time (tens of seconds), which limits their suitability to applications where the sensor is exposed to rapid changes of the gas concentration. Our proposal consists of exploiting a double first-order model of the MOX-based sensor from which a steady-state output is anticipated in real time given measurements of the transient state signal. This approach assumes that the nature of the volatile is known and requires a precalibration of the system time constants for each substance, an issue that is also described in the paper. The applicability of the proposed approach is validated with several experiments in real, uncontrolled scenarios with a mobile robot bearing an e-nose.

## 1. Introduction

The deployment of olfactory sensors is becoming an increasing practice in many industrial and environmental applications due to advances in the gas sensing technology. The exploitation of olfactory sensors can be classified into two main groups according to the level of control over the measurement conditions: Closed Sampling Systems (CSS), where the gas sensors are usually hosted in test chambers with controlled airflow, volatile exposure times, temperature and humidity, *etc.*, and Open Sampling Systems (OSS), with no control over the sensing conditions. Our interest is in the latter, which are more flexible and practical for field applications. Examples of such uses are environmental exploration [1], gas distribution modeling [2], buried land mine detection [3] or pollution monitoring [4]. Some of these applications are usually accomplished with the help of a mobile robot carrying the sensors on board, which makes the sensing task even more challenging.

Within the different technologies and materials available for gas sensor fabrication [5], MOX (Metal Oxide Semiconductor) transducers are one of the most popular and widely employed in mobile robotics olfaction, due to their high sensitivity and low prices. However they present some shortcomings including poor selectivity, response drift (age factor), influence by environmental factors such as humidity and temperature [6] and major limitations in their response speed [7]. These limitations come from the sensing mechanism underlying MOX technology, that is, the exchange of oxygen molecules between the volatile and the metal film [8,9].

**Figure 1.**
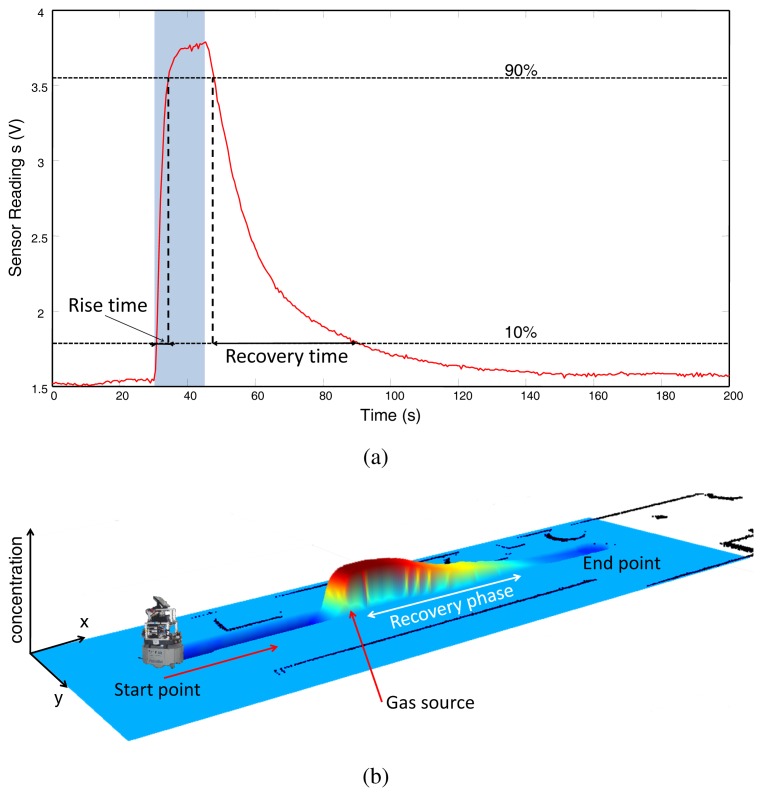
Rise and recovery phases of MOX sensor response to a step gas concentration. Figure (a) shows a 2D plot of the sensor response over time. The shaded blue region denotes the sensor exposure to the analyte. Figure (b) depicts a 3D gas distribution map generated from the reading of an MOX sensor carried by a mobile robot along a corridor. Observe how the recovery phase after the gas exposure is several time longer than the rise one.

Among these drawbacks, the long duration of the acquisition cycles (up to tens of seconds) is of special concern for OSS, since inaccurate readings are inevitable when measuring rapid changes of gas concentration, as illustrated in Figure 1. Observe how this limitation is particularly noticeable in the decay phase, when the output recovers to the baseline level (the steady output value given by a gas sensor when exposed to clean air). As a consequence of this slow dynamic response and because of the intermittent and chaotic nature of turbulent airflow in OSS [10,11], steady state values are rarely reached, and therefore gas sensing based on MOX technology must deal with the transient information of the signals [12]. This problem becomes crucial when the sensors are carried on a vehicle (typically a mobile robot) to provide measurements along the way. The adopted solution in such cases is to reduce the vehicle velocity to a few cm/s, such in [13]. This proposal, however, is not acceptable in many applications since the sampling of space must be as quickly as possible to cope with the rapid dynamics intrinsic to gas propagation.

To overcome this shortcoming of MOX-based electronic noses, we propose to estimate the steady state sensor output from the noisy and distorted transient signal, which corresponds to the search for an inverse dynamical model. In this work we follow the method to obtain, first, a forward model of the system and then invert this model. For that, we rely on a linear sensor model that comprises two first-order systems corresponding to the rise and recovery phases, respectively, with variable time constants. The applicability of the proposed approach to Open Sampling Systems is validated through three different experiments in real scenarios, performed with a mobile robot bearing an MOX-based e-nose.

## 2. Related Works

In general, a model seeks to represent a system (empirical objects, phenomena, and physical processes) in a logical, objective and simplified way, allowing to predict the output of the system provided the input. Thus, a model of an MOX sensor must predict the sensor resistance (transient and steady state) when exposed to a certain gas concentration profile. Our interest in having such a model is to use it in a reverse way: given a sequence of measurements from the transient response of the MOX sensor, we seek the exciting gas distribution through the estimation of the steady state sensor resistance.

The modeling of the dynamics of MOX sensors has been addressed in the literature for a variety of purposes. Gardner *et al*. [14] proposed a non-linear diffusion-reaction model to obtain the theoretical transient and steady state responses based on the reactions taking place at semiconductor level. This model is not applicable to our approach since we pretend to obtain the gas distribution from the sensor readings rather than from the electrical and physical properties of the sensors. Later, aiming at increasing the response speed of gas sensors, T. Yamanaka *et al.* [15] reported a two-phases (corresponding to the rise and decay phases) second order linear model to describe the transient response of a semiconductor gas sensor from a visualized gas distribution image. Despite its success, this model requires the use of a CCD camera as a gas detector, which is neither our case.

More recently, E. Llobet [16] reviewed the principal methods for dynamic analysis of the gas sensor response. Interestingly, the main use of these methods is to perform gas classification based on the transient response, from which a feature vector is extracted. For example, in [17] Box-Jenkins linear filters were applied to model an array of MOX sensors in the presence of four alcohols and water vapor with the aim of reducing the effect of the sensor drift in a classification process, and in [18] a multi-exponential transient spectroscopy (METS) method is proposed to improve the selectivity of chemical sensors in the analysis of gas mixtures.

Special mention deserves some works that rely, as it is our case, on modeling the sensor response to predict steady state values from the initial part of the transient. In [19], a so-called ARMA and multi-exponential models are proposed for reducing the time necessary to calibrate a sensor array. Nevertheless, since the focus is on the calibration of MOX sensors, the dynamic models are only applied to the rise transient signals recorded in Closed Sampling Systems over long time periods (over 800 s), while we aim to predict the gas distribution profile in real time and in OSS. In [20], A. Pardo *et al.* propose and compare different nonlinear inverse dynamic models of gas sensing systems for quantitative measurements. However, the considered dynamic conditions differ from those of OSS. First, a measurement chamber is used to obtain the gas sensor readings, which implicitly modifies the dynamic properties of the measured signals, and second, the acquisition frequency is too low (one sample per minute) to reflect the fast and highly dynamic changes of the gas concentration in OSS.

**Figure 2.**
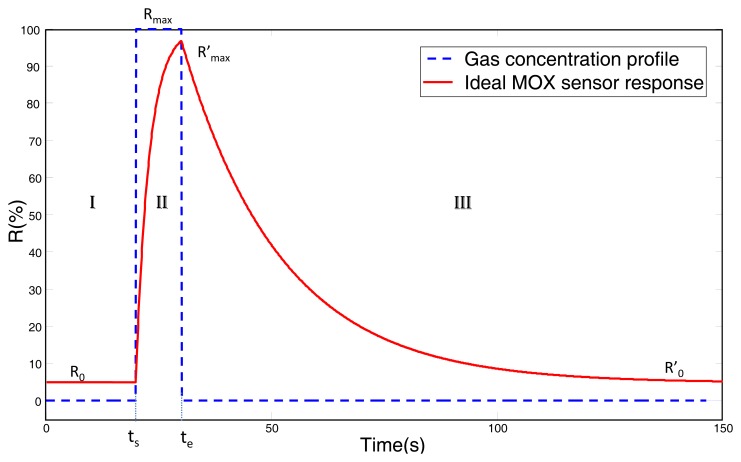
Ideal response of an MOX sensor (solid red line) when excited with a step gas concentratration (dashed blue line). The curve shows the three phases of a measurement: (I) baseline, (II) GAS measurement, and (III) recovery phase.

Based on the multi-exponential model proposed elsewhere [19,21], and taking into account the differences between rise and decay phases of MOX sensors, in this work we exploit a simplified version of it, where only one exponential is considered to model each phase (see Figure 2). According to this model, mathematically expressed in Equation (1), three phases can be considered in the output of a typical MOX gas sensor when exposed to an ideal step in concentration: baseline, gas measurement (rise) and recovery.

(1) $$R(t)=\{\begin{array}{ll} R_{0} & ,t<t_{s}\\ R_{0}+(R_{\max}-R_{0})\left(1-e^{\frac{-(t-t_{s})}{\tau_{r}}}\right) & ,t_{s}<t<t_{e}\\ R_{0}^{\prime }+(R_{\max}^{\prime }-R_{0}^{\prime })e\frac{-(t-t_{s}-\Delta t)}{\tau_{d}} & ,t_{e}<t \end{array}$$

where *τ_r_* and *τ_d_* are the time constants for the rise and recovery phases respectively, *t_s_* and *t_e_* represent the starting and ending times of the step excitation, *R*_0_ and $R_{0}^{\prime }$ are the sensor response level before and after the stimulus, *R_max_* is the saturation level, and $R_{\text{max}}^{\prime }$ is the maximum response level during the gas measurement phase. Notice that $R_{\text{max}}^{\prime }$ is usually lower than *R_max_* for short input pulses, as depicted in Figure 2.

This model was used by [22] for characterizing the response of an MOX-based e-nose carried by a robot. In this work we also exploit this model but making use of its inverted form, that is, to predict the gas distribution that the sensor is exposed to from its readings.

## 3. The Proposed MOX Model

**Figure 3.**
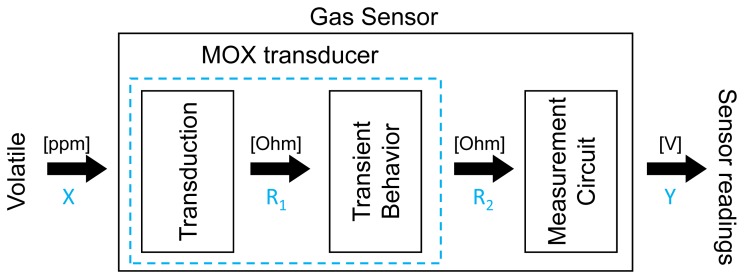
Block diagram of a smelling process with an MOX gas sensor. The sensor is excited by a volatile [ppm] producing a variation in the sensor resistance that is measured as an electrical signal [V] by means of a measurement circuit.

As shown in the block diagram of Figure 3, three different sub-processes can be distinguished in an MOX-based gas sensing process:

1. A non-linear static system representing the measurement electronic circuit (Ω to *V*).
2. A transformation (Ω to Ω) that captures the non-linear rate at which the sensor resistance varies over time (although we have separated the transduction and dynamic phases to explicitly denote both functionalities, both stages take place within the MOX transducer and they are most likely coupled).
3. A signal transduction mechanism (ppm to Ω) which results from the chemical interaction between the sensor sensitive surface and the molecules of reducing gases.

Next, we model each of these stages and invert them to come up with a complete inverted MOX sensor model. It is important to remark that the proposed inverted model does not aim to recover the gas concentration (X[ppm]), which corresponds to a sensor calibration problem not addressed here, but only the gas distribution, providing relative results proportional to the gas concentration (*R*_1_).

### 3.1. Measurement Circuit

This stage stands for the electronic circuit in charge of measuring the changes in the sensor resistance, which typically consists of a simple voltage divider:

(2) $$Y=V_{RL}(t)=\frac{V_{\text{CC}}\times R_{L}}{R_{2}(t)+R_{L}}$$

where *V_CC_* is the circuit voltage and *R_L_* is the load resistance.

Since the magnitude measured is the output voltage *Y* (*i*), we can easily recover the MOX resistance changes *R*_2_(*i*) by solving Equation (3).

(3) $$R_{2}(i)=\frac{R_{L}\times (V_{\text{CC}}-Y(i))}{Y(i)}$$

### 3.2. Transient Behavior Stage

As reported by previous authors [7,22], the transient response of an MOX sensor can be expressed by two first-order systems, as depicted in Equation (1). It clearly resembles a low-pass filter response with the particularity that the filter cutoff frequency $\left(f_{c}=\frac{1}{2\pi \tau }\right)$ is different for each phase the sensor is working at (rise or recovery). Equation (4) presents the two phases transfer function of this stage in the Laplace domain, where a common static delay (*t_d_*) has additionally been considered in both phases to compensate for the delay introduced by the pneumatic circuit used to draw in the gas and flow it through the sensors:

(4) $$\frac{R_{2}(s)}{R_{1}(s)}=\{\begin{array}{ll} \frac{A}{\tau_{r}s+1}e^{-t_{d}s} & ,\;\text{for rise phases}\;\\ \frac{A}{\tau_{d}s+1}e^{-t_{d}s} & ,\;\text{for recovery phases}\; \end{array}$$

where *s* is the Laplace variable, *A* is the filter gain, *τ_r_andτ_d_* are the filter time constants in the rise or recovery phase respectively, and *t_d_* (in seconds) is the system delay. For nomenclature clarification *τ_recovery_* has been denoted as *τ_d_*, where the subindex *d* stands for decay.

Since our interest remains in estimating the volatile distribution that the sensor is being exposed to, given the sensor readings, we work out *R*_1_ as a function of the sensor resistance measurements *R*_2_ by applying the inverse Laplace transform to Equation (4). As the transfer function has different expressions according to the phase (rise or recovery) that the sensor is working at, the parameters of the resulting differential equation will have to switch accordingly. Approximating the derivative of the measured sensor resistance $R_{2}^{\prime }$ by a backward first order finite difference, such differential equation can be written as:

(5) $$R_{1}(i-N)\propto R_{2}(i)+\tau \frac{R_{2}(i)-R_{2}(i-1)}{\Delta t}$$

where *R*_1_(*i*) is the unknown steady resistance value for the given gas concentration at the time step *i*, *R*_2_ is the measured sensor resistance, *τ* is the time constant for either the rise (*τ_r_*) or the recovery phase (*τ_d_*), is the number of samples for the system delay, and Δ*t* is the time between samples. Notice also that the scale factor *A* from Equation (4) has been dropped.

As observed in Equation (5), at each time step this dynamic model requires to know the value of parameters *τ_r_* and *τ_d_*, and the phase the sensor is working at. The latter may be determined from the slope of the measured MOX resistance *R*_2_, considering that the sensor is working under rise phase for positive values of the derivative, and under decay phase for negative values of it.

Notice that instead of working with the sensor resistance (which is inversely proportional to the volatile concentration), it may be more convenient to deal with the sensor conductance (*G_MOX_* = 1/*R_MOX_*), which is proportional to the gas concentration.

For estimating the values of *τ_r_* and *τ_d_*, a common practice in system modelling is that of identifying the parameters of the system upon its step response [23]. The problem with such procedure is that the time constants, in practice, depend not only on the type of volatile but also on its concentration [20]. In this paper this dependency is made explicit by adjusting a polynomial regression model over a sequence of concentration pulses with different amplitudes for each target gas. In Section 5.1, an example of such relation is depicted for the target gas ethanol.

### 3.3. Transduction Stage

The transduction stage is commonly defined by the sensitivity characteristics and the temperature and humidity dependencies of the transducer. For the case of Closed Sampling Systems, those characteristics are usually provided by the sensor manufacturer, relating the volatile concentration [ppm] to the sensor resistance ratio *R_S_/R*_0_ (sensor resistance in gas over sensor resistance in air) for different target gases and test conditions. Nevertheless, those sensitivity characteristics are obtained by measuring steady state values of the MOX sensor resistance after very long and constant exposure times, which are not applicable to Open Sampling Systems and thus not considered in this paper.

## 4. Signal Conditioning and Preprocessing

**Figure 4.**
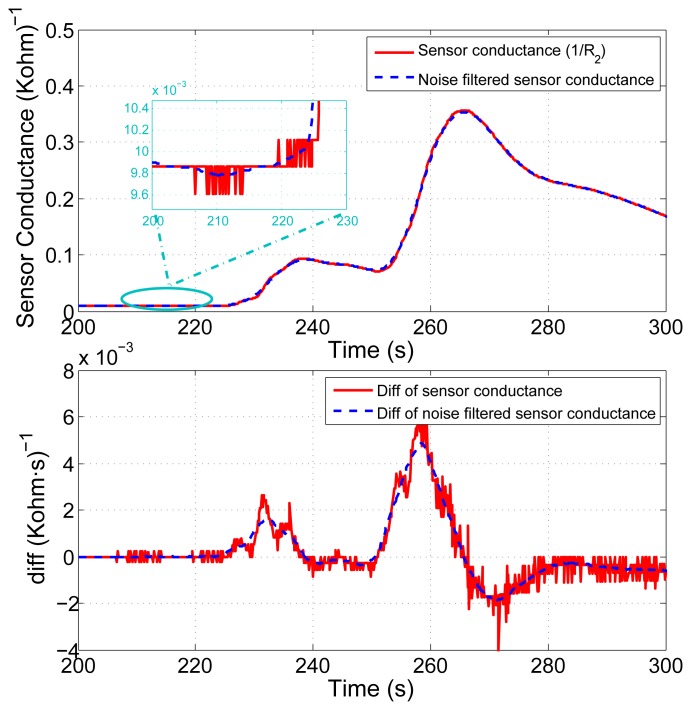
Noisy sensor conductance readings and its derivative (solid red line), and the corresponding filtered versions (dashed blue line).

The proposed model-based approach given by Equation (3) and Equation (5) relies on the sensor readings to obtain an estimation of the gas. As can be appreciated, a first order derivative needs to be computed to obtain such estimation, which notably degrades the signal-to-noise ratio and consequently the accuracy in the estimation (see Figure 4). Additionally, MOX sensors are susceptible to long and short term drift [5], gradually changing the sensor resistance even if exposed to exactly the same gas concentration under identical environmental conditions.

It then becomes necessary to carry out a signal conditioning to prepare the sensor readings to the posterior estimation process. Initially, for the purpose of drift compensation and dynamic range enhancement, the raw sensor readings *R*_2_(*i*) are divided by the sensor baseline resistance at *t* = 0, that is, *R*_2_(0). This transformation is known as relative baseline manipulation [5]. Later, in order to mitigate the noise effects on the model, a low pass filter followed by a sub-sampling process are applied to the signal, as depicted in Figure 4. The cutoff frequency of the filter and the down-sampling rate have been determined experimentally according to the sampling frequency.

## 5. Experimental Results

This section presents three different experiments designed with increasing complexity to test how the proposed model can anticipate the steady state values of the sensor resistance from transient measurements in Open Sampling Systems. We start by testing our approach in a scenario where airflow and volatile distribution were well controlled. Then, two experiments of gas distribution mapping in unmodified environments are described.

### 5.1. Train of Gas Pulses in a Controlled Scenario

This experiment is designed to validate, in the simplest possible way, the ability of the proposed approach, given by Equation (3) and Equation (5), to estimate the volatile distribution that the MOX gas sensors are being exposed to.

**Figure 5.**
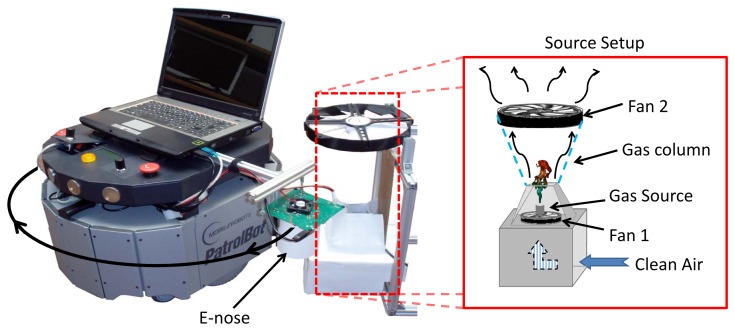
Picture of the Patrolbot mobile robot, the e-nose and the scheme of the gas source setup.

Since knowing the ground truth distribution of a volatile in an OSS is a tough task (we can even say utopian), a specific setup was designed to keep its concentration as constant as possible and to confine it in a predefined region, avoiding the dispersion of gas particles to undesired locations. The experiment setup is depicted in Figure 5. The gas source, composed by a small cup filled with acetone, was placed inside a cardboard box with a small upper aperture. A fan located at the bottom of the box (beneath the gas source) was used to generate a constant upstream airflow pushing the volatile through the chamber aperture, while a second fan, placed about 20 cm over the box, sucked up the air from the box. This setup allows us to keep the volatile confined in an approximated gas column between both fans.

A Patrolbot robotic base [24] was then placed in front of the gas source and commanded to rotate with constant angular speed *ω*. The e-nose, placed at the right side of the mobile base, then described a circular trajectory being exposed to the volatile only when passing through the gas column. The resulting excitation signal was then a train of identical duration pulses. The position of the source at each lap (and consequently the duration of each excitation pulse) was precisely obtained from the radial scan provided by a laser range scanner (SICK LMS500) carried by the Patrolbot.

Strictly speaking, because the sensor needs some time to entirely enter into the odor column, the excitation signal will not be a perfect pulse, but the deviation is small and can be neglected.

**Figure 6.**
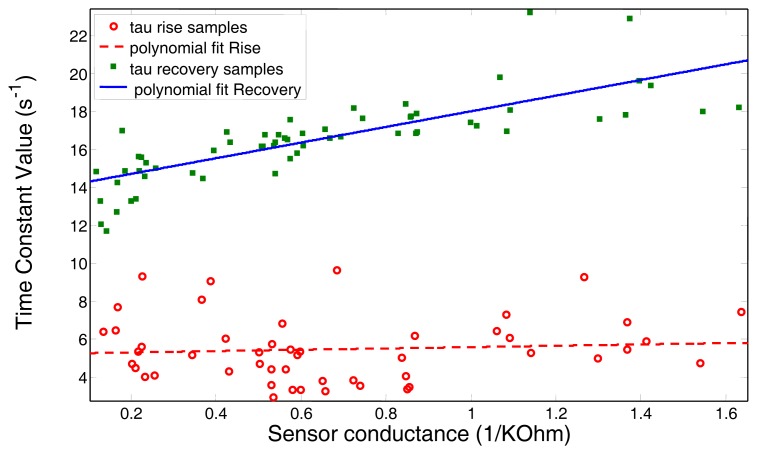
Pre-calibration of the time constants *τ_r_* and *τ_d_* with the gas concentration (*i.e.*, sensor conductance). Red circles and green squares represent the values of *τ_r_* and *τ_d_* respectively, while the lines represent their liner regression.

The pre-calibration of the time constants with the gas concentration, as depicted in Section 3.2, has been achieved by a polynomial regression over more than 50 short pulses with different amplitudes of the same volatile, in this case ethanol. The resulting dependency is depicted in Figure 6. Please, note that since the gas concentration is not available, we are considering the sensor conductance (1*/R*_2_) instead.

**Figure 7.**
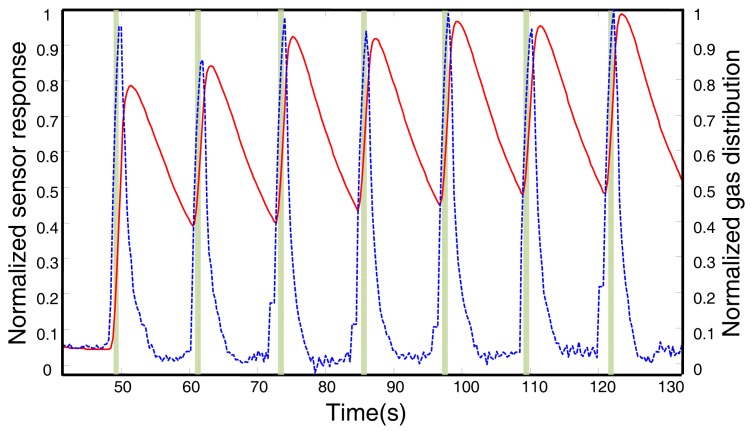
Results of the experiment in the controlled scenario for a robot angular speed *ω* = 30°/*s*. The train of acetone pulses (ground truth) is plotted as green shaded bars, the normalized raw sensor readings (Y) (See Figure 3) are plotted as a solid red line and the gas distribution estimated by the proposed approach is plotted as a dashed blue line.

Figure 7 presents the results of this experiment for a robot angular speed of 30°/*s*, that is, the e-nose is exposed to the same pulse every 12 s. The width of the pulse (in seconds) is computed from the robot rotational speed and the angular references detected from the laser scan. The sequence of rise and recovery phases in the sensor output (solid red line) can be observed. It is also clear how the long tails of the recovery phases are interrupted by the next rising phase. (This phase overlap produces an interesting accumulation effect in the sensor output, increasing the sensor reading value on each exposition to the gas source even though the volatile concentration was constant along the experiment duration. The study of this effect is out of the scope of this work.)

As can be noticed, the estimation of the gas distribution (dashed blue line) as provided by applying the proposed model is a more accurate estimation of the train of pulses than the raw sensor readings. The improvement is significant for the recovering phases, not only because the overlap between phases is avoided (the recovery phase is interrupted by the next rise phase before reaching the baseline), but because our estimation provides values of the gas distribution more consistent with the reality.

### 5.2. 1D Gas Distribution Mapping

The motivation of this experiment is to demonstrate the utility of the proposed approach in the generation of gas distribution maps, a challenging problem in robotic olfaction [25,26].

As described in the introduction, the slow recovery of MOX sensors becomes a serious drawback when the sensors are carried on a vehicle (typically a mobile robot). In such cases, the adopted solution is to reduce the vehicle velocity to a few cm/s, increasing considerably the execution time of the task. We demonstrate here that our estimation of the gas distribution to a certain extent removes the need to reduce the robot velocity for maintaining the map accuracy.

**Figure 8.**
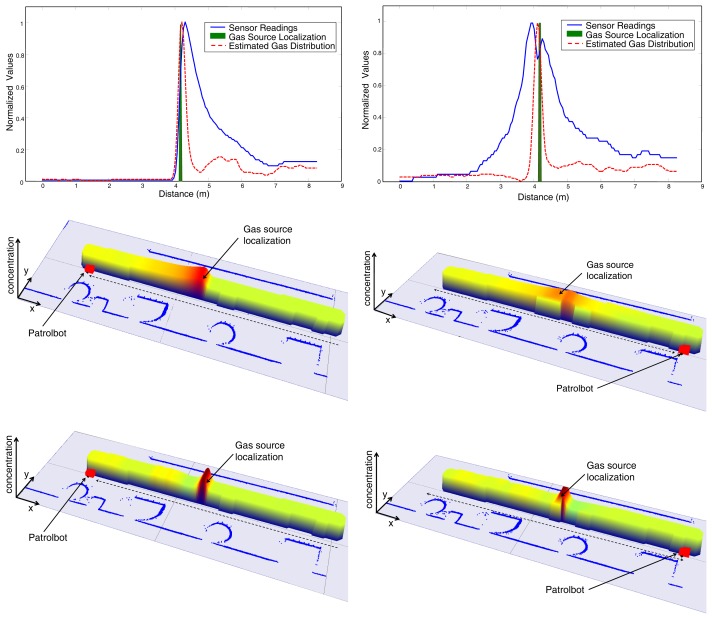
Snapshots of the maps generated in the 1D gas distribution mapping experiment at the middle and end of the robot path (left/right column, respectively), for a robot speed of 0.1*m/s*. Top row sub-figures illustrate a comparison between the normalized sensor readings and the gas distribution estimated here. Middle and bottom row sub-figures show a 3D reconstruction of the maps generated with the raw MOX readings (middle row), or the estimation provided by the proposed model (bottom row).

**Figure 9.**
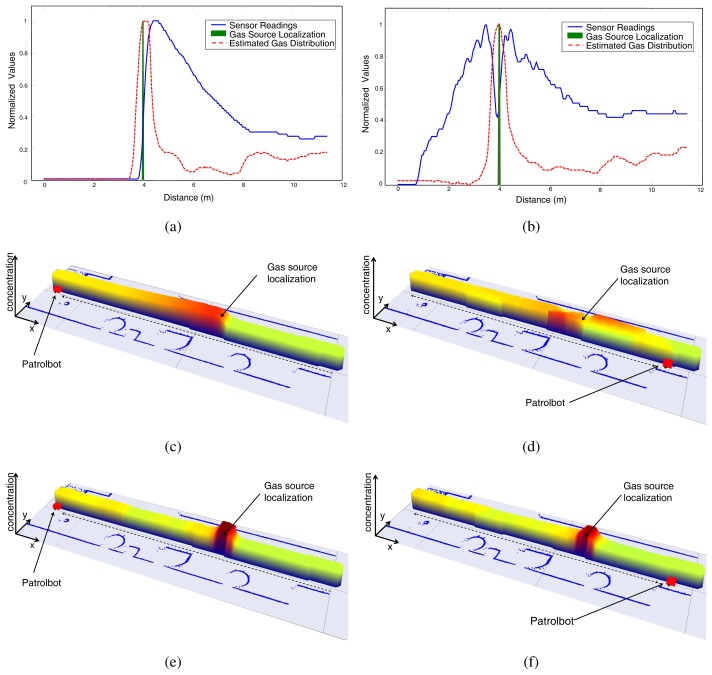
Snapshots of the maps generated in the 1D gas distribution mapping experiment at the middle and end of the robot path (left/right column, respectively), for a robot speed of 0.4*m/s*. Top row sub-figures illustrate a comparison between the normalized sensor readings and the gas distribution estimated here. The middle and bottom row sub-figures show a 3D reconstruction of the maps generated with the raw MOX readings (middle row), or the estimation provided by the proposed model (bottom row).

For such a goal, two different gas distribution maps of an unmodified long indoor corridor are generated by driving the robot at two different velocities. Figure 8 shows a comparison of the gas maps generated using the Kernel-based method proposed in [27] for a reduced robot speed of 0.1 m/s, while Figure 9 represents the results of a similar experiment using a robot speed of 0.4 m/s.

In both cases, the robot travels the corridor twice (round trip), passing over a cap filled with acetone placed in the middle of it. The map evolves as the robot moves and collects new sensory data, so we decided to analyze the map as built, at two different points of the robot path: at the end of the corridor (top row sub-figures), and at the initial position after the task has been completed (bottom row sub-figures).

As can be seen from the results, the “*tail*” effect produced by the slow recovery of MOX sensors leads to a gas distribution map with high concentrations along the robot path once the source has been hit. As expected, this effect is more harmful when increasing the robot speed, one of the reasons why the speed of olfactory robots is usually kept small. Please, note how this effect is substantially palliated when applying the proposed gas estimation (see Figure 9(e) and Figure 9(f)), even when the robot speed is high.

As a conclusion, our approach not only presents a noticeable improvement in the correct localization of the gas source but also provides confident values of the gas distribution along the robot path. This is important since in most cases it is desirable to not only know the location of the gas source but also know how the volatile emanating from it has spread in the surroundings.

### 5.3. 2D Gas Distribution Mapping

In this last experiment, we pursue to test our model-based estimation of the gas concentration in a more complex environment. The idea is to accomplish a complete olfaction mission in a realistic scenario where no alterations of the environment are done. Typical examples are the localization of leaks or the declaration of areas of high concentration levels of harmful gases.

**Figure 10.**
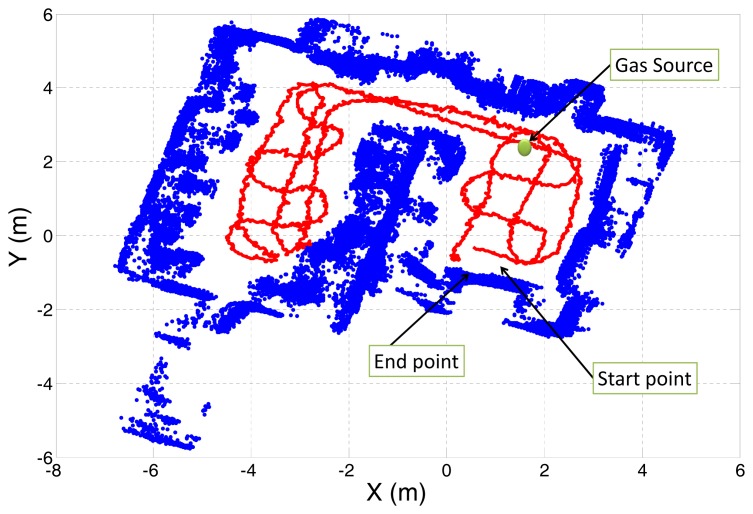
Map of the experimental area used in the 2D mapping experiment. Blue points represent obstacles detected by the onboard SICK laser scan, the robot path is marked as red solid line, and the gas source position is pointed with a green circle.

The testing scenario consisted of two adjacent rooms communicated through a small corridor (see Figure 10). The robot inspected both rooms following the predefined path marked as solid red line, at a speed of 0.3*m/s*. The gas source, composed by a cardboard plate impregnated in ethanol, was placed on the floor along the robot path. To be able to compare the maps generated from the raw sensor readings with those provided after applying the proposed MOX sensor model, doors and windows were kept closed to avoid uncontrolled airflows.

**Figure 11.**
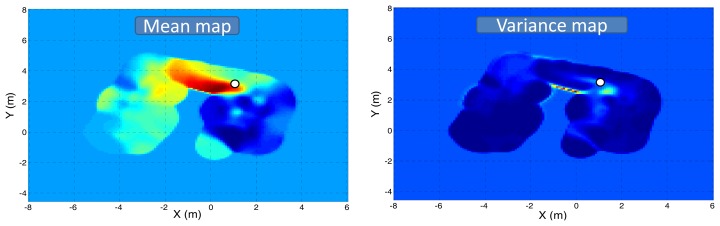
Mean and predictive variance gas distribution maps of the inspected area generated by the KernelDM+V algorithm when fed with the sensor readings (after baseline manipulation and delay correction). The gas source location has been marked as a white circle.

**Figure 12.**
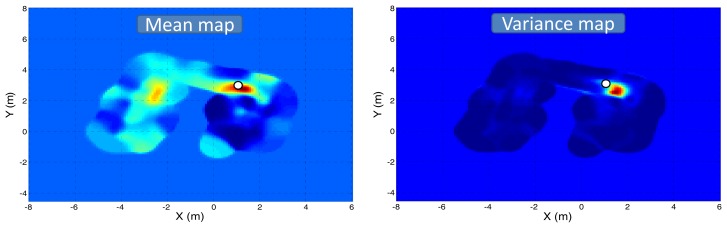
Mean and predictive variance s distribution maps of the inspected area generated by the KernelDM+V algorithm after applying the proposed model-base estimation of the gas concentration. The gas source location has been marked as a white circle.

Figures 11 and 12 depict the mean and predictive variance gas distribution maps generated by the KernelDM+V algorithm [2], when fed with the MOX sensor readings and the model-based gas estimation, respectively.

Focusing on the mean maps, it can be seen that in both cases the maximum concentration falls near the real source location (marked as a white circle). However, when the map is built from the raw sensor readings, a high concentration area appears along the corridor connecting both rooms. The shape reassembles that of a gas plume generated by a dominant airflow, which we know is not possible since doors and windows were kept closed during the experiment. This “fake” plume is attributed to the actual robot path and the slow recovery of MOX sensors. When building the map from the gas concentration estimated by the proposed model (Figure 12), this “fake” plume does not appear, correctly representing the gas distribution in the inspected area.

Focusing now on the predictive variance maps, it has been previously reported that the variance of a set of gas concentration measurements has been suggested as a feature that can identify the location of a source of gas [27,28]. Following this principle, it can be seen that the gas source cannot be precisely located in the first case (Figure 11) since there are high variance values not only nearby the real location of the source but also along the corridor. Again, the application of the proposed MOX model leads to a predictive variance map where all high variance values fall near the real source location, allowing a correct estimation of it.

## 6. Conclusions and Future Works

In this paper, a modeling approach has been reported for the improvement of open sampling system olfaction applications based on MOX gas sensors. The proposed approach compensates the slow dynamic behavior of the MOX sensor, by forecasting the steady state values of the sensor resistance from a sequence of transient measurements. The exploited model is based on two first order systems (rise and recovery) with time constants that depend on the sensor reading amplitude. Our approach has been validated in different scenarios, demonstrating its utility in applications like gas source localization or gas distribution mapping. Additionally, we have proved that our approach enables a considerable increase in the speed at which a mobile base carrying the MOX-based e-nose can inspect the environment, which directly implies an important reduction in the execution times of the olfaction task.

Future work includes modeling additional parameters as temperature and humidity in order to improve the gas distribution estimation in real complex scenarios. Besides, the study of the transduction stage (calibration) for open sampling systems will be considered to complete the MOX sensor model.
